# Prophylactic mesh placement to avoid incisional hernias after stoma reversal: a systematic review and meta-analysis

**DOI:** 10.1007/s10029-019-01996-8

**Published:** 2019-07-13

**Authors:** L. C. L. van den Hil, S. van Steensel, M. H. F. Schreinemacher, N. D. Bouvy

**Affiliations:** 1 Department of General Surgery, Maastricht University Medical Centre, Maastricht, 6202 AZ The Netherlands; 2 NUTRIM School of Nutrition and Translational Research in Metabolism, Maastricht University, Maastricht, 6200 MD The Netherlands; 3 Department of General Surgery, Maastricht University Medical Centre, P.O. Box 616, 6200 MD Maastricht, The Netherlands

**Keywords:** Temporary stoma, Stoma reversal, Incisional hernia, Prophylactic mesh

## Abstract

**Purpose:**

To provide an overview of the available literature on prevention of incisional hernias after stoma reversal, with the use of prophylactic meshes.

**Methods:**

A literature search of Pubmed, MEDLINE and EMBASE was performed. Search terms for stoma, enterostomy, mesh, prophylaxis and hernia were used. Search was updated to December 31th 2018. No time limitations were used, while English, Geman, Dutch and French were used as language restrictions. The primary outcome was the incidence of incisional hernia formation after stoma reversal. Secondary outcomes were mesh-related complications. Data on study design, sample size, patient characteristics, stoma and mesh characteristics, duration of follow-up and outcomes were extracted from the included articles.

**Results:**

A number of 241 articles were identified and three studies with 536 patients were included. A prophylactic mesh was placed in 168 patients to prevent incisional hernias after stoma reversal. Follow-up ranged from 10 to 21 months. The risk of incisional hernia in case of prophylactic mesh placement was significantly lower in comparison to no mesh placement (OR 0.10, 95% CI 0.04–0.27, *p* < 0.001, *I*^2^ = 0%, CI 0–91.40%). No differences in surgical site infections were detected between the groups.

**Conclusions:**

The use of a prophylactic mesh seems to reduce the risk on incisional hernias after stoma reversal and therefore mesh reinforcement should be considered after stoma reversal.

**Electronic supplementary material:**

The online version of this article (10.1007/s10029-019-01996-8) contains supplementary material, which is available to authorized users.

## Introduction

Stoma formation is most frequently performed for colorectal cancer, followed by both diverticular disease and inflammatory bowel disease [1]. In up to 30% of the patients with colorectal cancer the stoma is permanent [2]. Temporary stomas are constructed mainly to protect distal colorectal anastomoses, aiming to prevent the consequences of leakage of the anastomosis [3]. Unfortunately, the complication rate after stoma formation is high with reported numbers up to 70% [4]. Complications which may occur during the presence of a stoma include a high output stoma, prolapse, stenosis, necrosis, fistulae, retraction and parastomal herniation [4, 5]. After stoma reversal, there is an increased risk to develop incisional hernias [1].

Parastomal hernias seem to occur more often in patients with a colostomy, compared to patients with an ileostomy. This might be caused by the fact that ileostomies are frequently temporary stomata. Ileostomies are intended to be reversed a few months after primary surgery and in this short period of time, less hernias may develop [6]. Yet, even after closure of a temporary stoma, incisional herniation at the stoma site may occur. This is an underestimated problem with a reported incidence of up to 30–48% depending on the diagnostic modality used [1, 7]. Incisional hernias can lead to pain, bowel obstruction and strangulation [6, 8, 9]. Therefore, 44% of the clinically relevant incisional hernias necessitated surgical repair with a mesh as well [1].

In the recent past, several studies regarding prophylactic mesh placement to prevent incisional hernias after laparotomy have shown promising results [10, 11]. Also prophylactic mesh placement around permanent stomata has widely been examined. Last year, multiple meta-analyses regarding this topic were published and showed that prophylactic mesh placement around a stoma is safe and reduces the number of parastomal hernias [12–14].

Although the risk on incisional hernias is high after reversal of a temporary stoma and thus using a prophylactic mesh during stoma reversal seems favourable, hardly no systematic reviews or meta-analyses could be found regarding this topic. Taking into account that up to 26% of all temporary stomata will not be reversed eventually, it seems even more likely that these stomata also deserve a prophylactic mesh during stoma formation in order to prevent stoma-related hernias [15].

Therefore, the aim of this study was to assess the effectiveness of prophylactic meshes in preventing incisional hernia at the site of stoma reversal.

## Methods

To perform an adequate literature search, the following PICO was formulated: In adults (*P*), does a prophylactic placed mesh (*I*), compared to conventional treatment without a mesh (*C*), decrease the risk on incisional hernia after stoma reversal (*O*)?

## Search

A literature search of Pubmed, MEDLINE and EMBASE was performed, according to the Preferred Reporting Items for Systematic Reviews and Meta-Analyses (PRISMA) guidelines [16]. Search terms and synonyms for stoma, enterostomy, mesh, prophylaxis and hernia were used as MeSH and free text terms. Table 1 shows the complete search. Subsequently, the reference lists of included articles and previous reviews were searched.

**Table 1:** Terms used in the literature search

Search terms			
Surgical stoma (MesH)	Surgical mesh (MesH)	Prophylaxis	Abdominal hernia (MesH)
Enterostomy (MesH)	Mesh	Prevention	Hernia
Stomas	Meshes	Preventive measures	Hernias
Stoma	Prosthesis	Preventive measure	Herniation
Stomata	Prosthetic	Preventive therapy	Herniations
Enterostomy		Control	Wall defect
Enterostomies		Prophylactic	Wall defects
Colostomy			
Colostomies			
Ileostomy			
Ileostomies			
Gastroenterostomy			
Gastroenterostomies			
Bowel continuation			
Digestive continuation			
Bowel restoration			
Digestive restoration			

## Study selection

Two independent reviewers screened all studies (LCLvdH, SvS). No time limitations were used. English, German, French and Dutch were used as language restrictions. The search was updated up to 31 December 2018. Articles that reported on incisional hernias after prophylactic mesh placement at the stoma site in humans older than 18 years were included. Studies without a control group were excluded for data analysis. Next, case reports (less than 10 patients), letters, animal studies, review articles and meta-analysis were excluded. If articles described the same study population, the most recent publication was used. Disagreements were resolved by re-examination of the involved studies until consensus between the reviewers was reached.

## Data extraction and study outcome

**Fig. 1:**
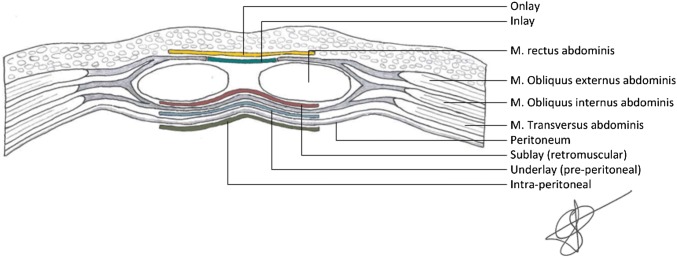
Different locations of mesh placement

The data were extracted and checked by two reviewers (LCLvdH, SvS). Data on study design, sample size, patient characteristics, stoma and mesh characteristics, duration of follow-up and outcomes were extracted from the included articles. Stoma and mesh characteristics include the type of surgery (laparotomy or laparoscopy), type of stoma (e.g., ileostomy, colostomy), type of mesh and type of mesh placement (e.g., onlay, sublay). See Fig. 1 for the different locations of mesh placement. The primary outcome was the incidence of incisional hernia formation at the former stoma place during follow-up. Secondary outcomes were mesh-related complications.

## Quality assessment

The risk of study bias was assessed by one reviewer (LCLvdH) and controlled by another (SvS), using the Methodological Index for Non-Randomized Studies (MINORS) [17]. This is a validated instrument to assess the degree of bias of non-randomized trials. It consists of 12 items which can be scored from 0 to 2; 0 indicating that the item was not reported, 1 that the item was reported inadequately, and 2 that the item was reported adequately.

## Data synthesis and analysis

A meta-analysis was performed for the primary outcome, the incidence of incisional hernia after stoma reversal. The Mantel–Haenszel method was used to calculate the effect on binary outcomes, which was expressed in pooled odds ratios with 95%-confidence intervals. Heterogeneity was expressed using the *I*^2^ statistic and the random effect model was applied. To confirm that the results of the present meta-analysis were not based on one single study, a leave-one-out analysis was performed.

A subgroup analyses ileostomy versus colostomy and the occurrence of surgical site infections were performed. Analyses were carried out with RevMan software version 5.3, provided by the Cochrane Collaboration [18].

## Results

**Fig. 2:**
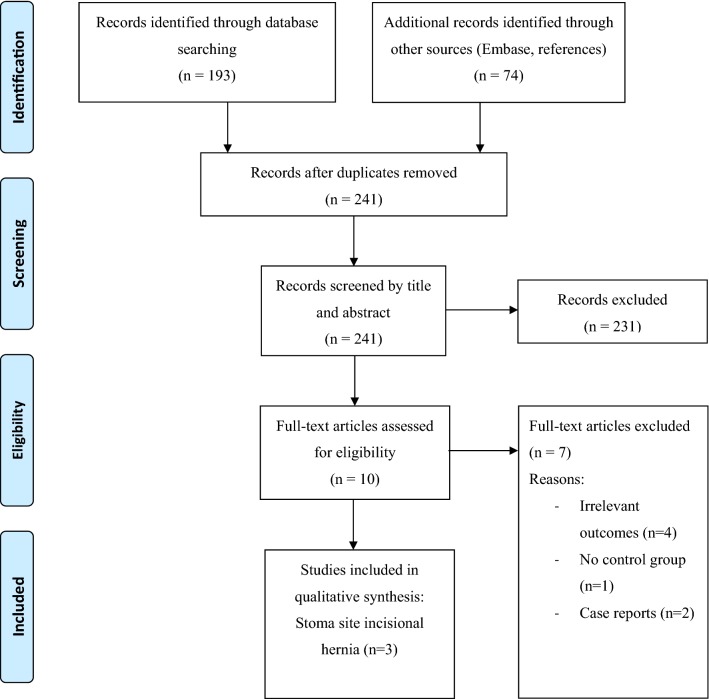
Flowchart of study inclusion

In total, a number of 241 articles were identified and screened. Duplicates and studies that did not mention our main outcome were excluded. A total of three studies with 536 patients were included [19–21]. The complete selection procedure is shown in Fig. 2.

## Quality assessment

The range on the MINOR index of the included studies varied from 15 to 20 points. All studies scored poorly on blinding of the researchers or participants. The time of follow-up was adequately mentioned in all included studies. The complete scores can be found in Table 2.

**Table 2:** Outcomes of methodological items for non-randomized studies

Items	Liu et al. [19]	Maggiori et al. [20]	Warren et al. [21]
A clearly stated aim	2	1	2
Inclusion of consecutive patients	2	2	0
Prospective collection of data	0	2	1
Endpoints appropriate to the aim of the study	1	1	2
Unbiased assessment of the study endpoint	1	1	0
Follow-up period appropriate to the aim of the study	2	2	1
Loss to follow up less than 5%	2	2	2
Prospective calculation of the study size	2	0	0
Additional criteria in the case of comparative study			
An adequate control group	2	2	2
Contemporary groups	2	1	2
Baseline equivalence of groups	2	2	1
Adequate statistical analyses	2	2	2
Total score	20	18	15

Items can be scored from 0–2, 0 if the item is not reported, 1 if the item is reported inadequately, 2 if the item is reported adequately

## Characteristics of included studies

Of the included studies, two had a retrospective design [19, 21] and one was a prospective, case-matched study [20]. Follow-up of the included studies ranged from 10 to 21 months. Table 3 shows the study and patient characteristics of the included studies.

**Table 3:** Study characteristics of the included studies

Author	Year	Design	Level of evidence	Study details		Patients			
				Group	Follow-up time (months)	Number	Sex (M, %)	Age	BMI
Liu et al. [19]	2013	R	2b	Control	21.1 (IQR 10.1–33.9)	36	21 (58.3%)	65.0 (IQR 57.8–70.5)	27.8 (5.3)
				Mesh	18.0 (IQR 13.8–26.2)	47	30 (63.8%)	69.6 (IQR 57.9–76.0)	25.6 (4.6)
Maggiori et al. [20]	2015	P	3b	Control	39.2 (16.9)	64	40 (62%)	61 (13)	25 (4)
				Mesh	16.8 (3.3)	30	18 (60%)	61 (13)	26 (4)
Warren et al. [21]	2017	R	2b	Control	14 (IQR 3–30)	268	146 (54.5%)	54.8 (15.7)	27.3 (6.4)
				Mesh	6.5 (IQR 2.25–14.75)	91	49 (54%)	57.3 (11.3)	30.2 (7.1)

Continuous data are median (interquartile range), median (range) or mean (standard deviation)

*R* retrospective study, *P* prospective study, *IQR* inter quartile range, *M* male

Out of 536 patients, 324 patients received an ileostomy. A colostomy was formed in the remaining 212 patients. In 168 patients a prophylactic mesh was placed and 368 patients were included in the control group. In all studies, antibiotics were administered peri-operatively. In the study of Warren et al. [21] antibiotics were administered locally. All studies described mesh placement during stoma reversal [19–21]. Table 4 shows more detailed information on stoma and mesh characteristics. Duration to stoma reversal differed between the studies, with a range of 6 weeks to 9 months. Warren et al. [21] did not mention the time between stoma creation and stoma reversal.

**Table 4:** Stoma and mesh characteristics described in the included studies

Author	Year	Group	Ostomy		Indication				Mesh type	Mesh fixation	Mesh location
			Ileostomy	Colostomy	CRC	IBD	Div	Other			
Liu et al. [19]	2013	Control	36	0	25	9*	6*	5*	N.A	N.A	N.A
		Mesh	47	0	38				Polypropylene	Sutures	Onlay
Maggiori et al. [20]	2015	Control	64	0	64	0	0	0	N.A	N.A	N.A
		Mesh	30	0	30	0	0	0	NCC PDM	Sutures	Sublay
Warren et al. [21]	2017	Control	123	145	N.S				N.A	N.A	N.A
		Mesh	24	67					Polypropylene	Sutures (*n* = 83) Glue (*n* = 2) No fixation (*n* = 6)	Sublay (*n* = 87) Underlay (*n* = 4)

*CRC* colorectal carcinoma, *IBD* inflammatory bowel disease, *Div* diverticular disease, *N.S.* not specified, *N.A.* not applicable, *NCC PDM* non-crosslinked collagen, porcine dermal matrix

*The numbers of patients per group with IBD, diverticulitis or other cause for ileostomy were not further specified in the study of Liu et al

During follow-up, physical examination and ultrasonography or CT-scans were performed to detect incisional hernias. One study described the definition of a hernia [20].

## Hernia rates

**Fig. 3:**
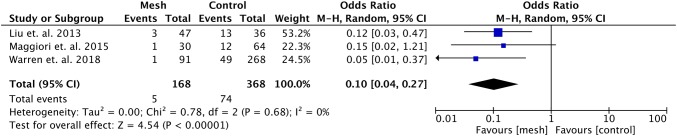
Forest plot of incidence of incisional hernias after stoma reversal

Three studies were included in the meta-analyses [19–21]. The risk of incisional hernia in case of prophylactic mesh placement was significantly lower in comparison to no mesh placement (OR 0.10, 95% CI 0.04–0.27, *p* < 0.001, *I*^2^ = 0%, 95% CI 0–91.40%). Figure 3 shows the corresponding forest plot.

The results of the leave-one-out sensitivity analysis were comparable, indicating that the results of this meta-analysis are not based at one single study (Supplementary Fig. 1).

**Fig. 4:**
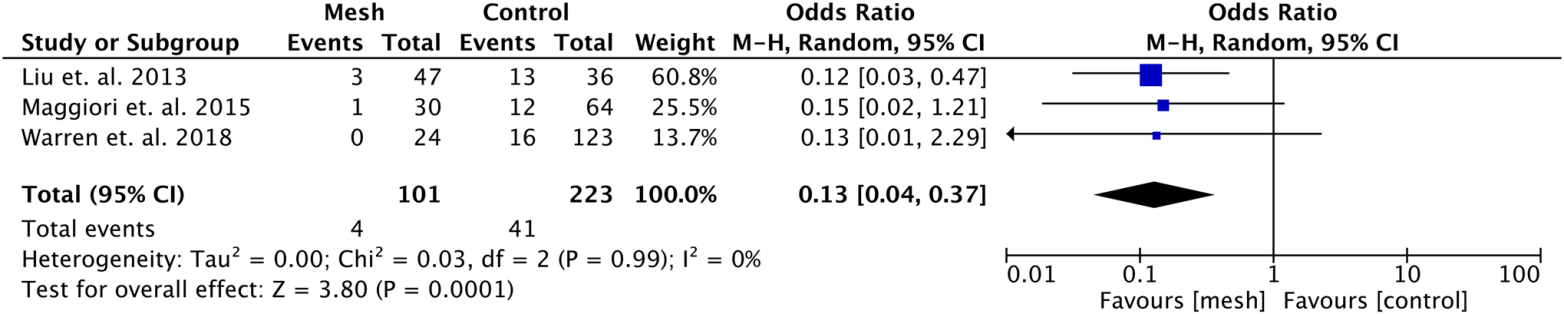
Forest plot of incidence of incisional hernias after ileostomy reversal

**Fig. 5:**
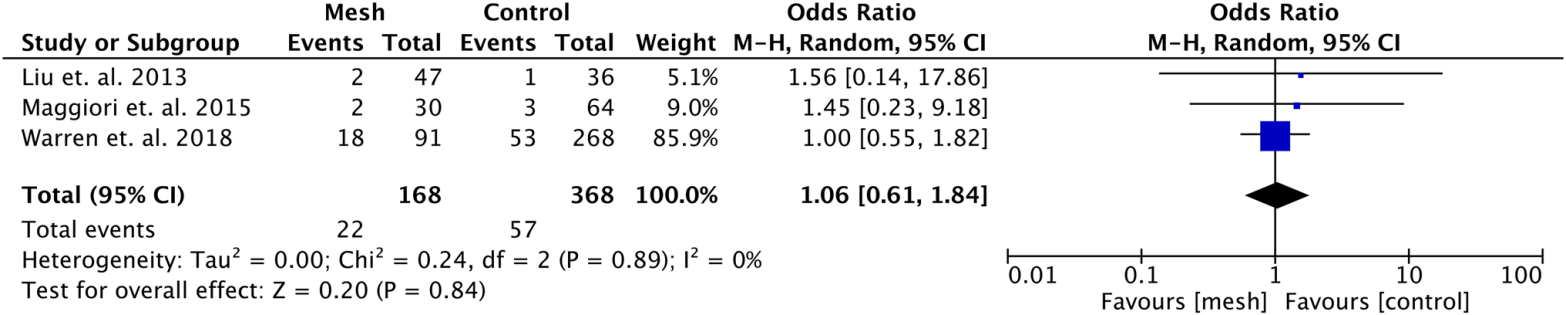
Forest plot of incidence of surgical site infections after stoma reversal

Warren et al. [21] described ileostomies separately from colostomies and was the only included study also reporting on colostomies. The data regarding the incidence of incisional hernia at the former ileostomy site were pooled in a subgroup analysis (see Fig. 4). The odds ratio was 0.13 (95% CI 0.04–0.37, *p* < 0.001, *I*^2^ = 0%) in favor of preventive mesh placement. Regarding surgical site infections, no significant differences were found comparing preventive mesh placement with no mesh placement (OR 1.06, 95% CI 0.61–1.84, *p* = 0.84, *I*^2^ = 0%) (see Fig. 5).

## Discussion

The current review and meta-analysis show that hernia rates after stoma reversal can decrease significantly when a prophylactic mesh is used. Although surgeons are reluctant to use synthetic materials in fear of mesh infection, no differences in risk of infection could be detected.

Studies with as primary outcome the incidence of hernias at the former stoma site reported incidences from 13 to 18% [22, 23]. This high percentage is comprehensible when the stoma site is considered to be a hernia that currently is closed primarily using the suture technique instead of mesh repair. Suture repair of hernias is nowadays obsolete due to the increased recurrence rates compared to mesh repair [24]. The necessity to reinforce former stoma sites with a mesh is underlined in the current study, although the numbers of included patients are small and the follow-up lengths are relatively short. Furthermore, only limited data regarding secondary outcomes are available. However, evidence in favour of prophylactic meshes to prevent midline incisional hernias and parastomal hernias is rising [10, 11, 14]. Thus, it seems advisable to use prophylactic meshes in patients at risk for developing incisional hernias, although studies regarding the quality of life and patient reported outcomes measures (PROMs) are needed to evaluate the overall benefits of prophylactic mesh placement after stoma reversal.

Risk factors for incisional hernias are increased age, obesity and connective tissue disorders. Other risk factors might be malnutrition, elevated intra-abdominal pressure and comorbidities that influence a normal wound healing [25]. Surgery-related risk factors are larger aperture size and peri-stomal complications, such as prolapse, obstruction or retraction [25, 26]. A recent meta-analysis has also shown an increased risk of incisional hernias after the closure of colostomies compared to ileostomies [27].

Next, the timing of mesh placement is of interest. In the included studies, meshes were placed during stoma reversal. However, in one pilot study that was not included in the analysis, meshes were placed at the time of temporary stoma formation [28]. An advantage of this technique is that there is a good ingrowth of the mesh at the time of stoma reversal and in case the stoma will not be reversed the mesh serves as a prophylaxis for parastomal hernia formation. No reversal of temporary stomata occurs in up to 26% of all temporary stomata. Therefore prophylactic mesh placement during stoma formation can have an extra benefit, namely to prevent both the occurrence of parastomal hernia and prolapse [15, 29]. On the other hand, mesh placement at the time of stoma creation might lead to problems when the stoma has to be dissected free from the surrounding tissue during stoma reversal [28].

Restraint of prophylactic mesh placement is required in an emergency setting. Operations in an emergency setting are thought to be more often complicated by contamination of the intra-abdominal cavity. This might lead to an increase in early postoperative complications, such as wound infections. A meta-analysis looking at the risk factors for mesh-related infections after hernia repair has shown that emergency hernia repair is a risk factor for mesh-related infections [30] and, therefore, caution is warranted regarding prophylactic mesh placement in these situations.

Some strengths and limitations of the current study need to be addressed.

The major strength of this study is its systematic approach and that it is the first meta-analysis published on the effect of prophylactic mesh placement during stoma reversal. It presents a good overview of the available literature.

On the other hand, data of high-quality studies are lacking, since no randomized controlled trials on this subject are available. Besides, we did not include grey literature or experts opinions.

Only three studies, whereof two retrospective studies and one prospective, case-matched study, were included. This may have led to selection bias. Second, the quality of included articles was moderate, mainly due to the design of the studies, namely non-randomized prospective or retrospective trials. Furthermore, adequate blinding was always lacking. Third, there was a considerable heterogeneity between the included studies. For example, meshes were placed in different layers of the abdominal wall. This might influence the development of hernias, although all included studies showed a lower risk of hernia formation when a prophylactic mesh was placed. In addition, a meta-analysis could not detect significant differences in recurrence rates after parastomal hernia repair with meshes placed in the different layers [31].

Next, both biological and synthetic meshes were used and within these groups also the materials differed. Currently, there is no evidence available that shows the superiority of synthetic meshes or biologic meshes regarding hernia recurrence rates and the use in contaminated fields [32, 33]. In addition, in the current study no differences in surgical site infections (SSI) were seen between the included studies using synthetic or biologic meshes. Regarding the synthetic meshes, in all studies low-weight meshes have been used, which probably decrease the risk on mesh infections [34].

Furthermore, the number of SSI did not differ between mesh and control groups. However, hernia occurrence instead of infection rates was reported as primary outcomes of the included studies. Therefore, it is uncertain whether the numbers of surgical site infections are accurate and thus conclusions should be drawn carefully.

Lastly, differences in follow-up time make it also more difficult to compare the studies in this meta-analysis. Follow-up lengths varied between 10 and 26 months. Although it is known that 75% of the hernias are present after two years, the prevalence of incisional hernias increases with a longer follow-up period [35]. Therefore, follow-up lengths in the included studies might be too short to draw hard conclusions regarding recurrence rates, although mesh-related infections most likely occur during the early follow-up period.

## Conclusion

All selected studies showed a reduced risk in hernia formation at the site of the stoma reversal when a prophylactic mesh was used. In addition, the reported lack of any mesh-related infection seems to justify the routine use of a prophylactic mesh during stoma reversal.

## Electronic supplementary material

Below is the link to the electronic supplementary material.

Supplementary file1 (DOCX 78 kb)
