# Supplementary material for: Prophylactic mesh placement to avoid incisional hernias after stoma reversal: a systematic review and meta-analysis
Source: Hernia. 2019 Jul 13;23(4):733–41. doi: 10.1007/s10029-019-01996-8 (PMC6661031; doi:10.1007/s10029-019-01996-8)

**
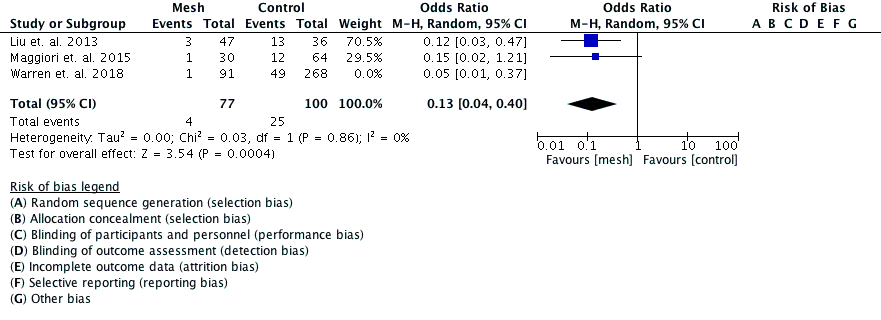
Supplementary figure 1.** Forest plot of leave-one-out analysis of incidence of incisional hernias after stoma reversal

**
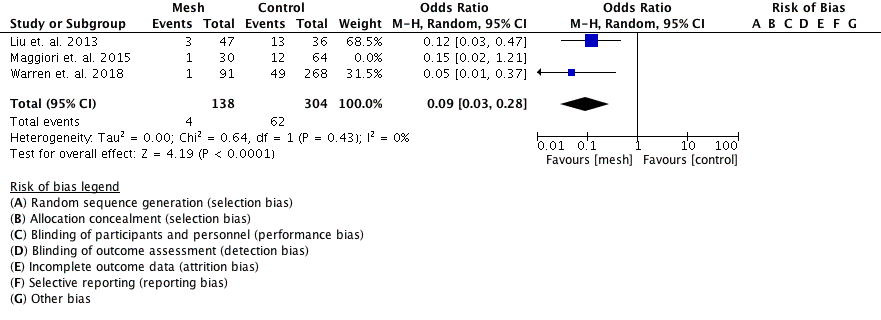
**


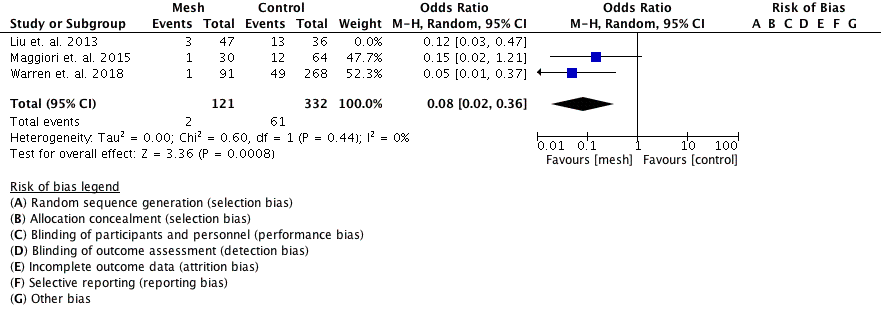

Supplement: Supplementary file 1 — Supplementary file1 (DOCX 78 kb) [file 10029_2019_1996_MOESM1_ESM.docx]
